# A pre-S gene chip to detect pre-S deletions in hepatitis B virus large surface antigen as a predictive marker for hepatoma risk in chronic hepatitis B virus carriers

**DOI:** 10.1186/1423-0127-16-84

**Published:** 2009-09-15

**Authors:** Fan-Ching Shen, Ih-Jen Su, Han-Chieh Wu, Yi-Hsuan Hsieh, Wei-Jen Yao, Kung-Chia Young, Tsung-Chuan Chang, Hui-Chuan Hsieh, Han-Ni Tsai, Wenya Huang

**Affiliations:** 1 Department of Medical Laboratory Science and Biotechnology, National Cheng Kung University, Tainan, Taiwan, Republic of China; 2 Institute of Basic Medicine, National Cheng Kung University, Tainan, Taiwan, Republic of China; 3 Center for Signal Transduction and Gene Regulation, National Cheng Kung University, Tainan, Taiwan, Republic of China; 4 Division of Infectious Diseases, National Health Research Institutes, Tainan, Taiwan, Republic of China; 5 Laboratory of Molecular Diagnostics, Department of Pathology, National Cheng Kung University Hospital, Tainan, Taiwan, Republic of China; 6 Department of Radiology, National Cheng Kung University Hospital, Tainan, Taiwan, Republic of China

## Abstract

**Background:**

Chronic hepatitis B virus (HBV) infection is an important cause of hepatocellular carcinoma (HCC) worldwide. The pre-S_1 _and -S_2 _mutant large HBV surface antigen (LHBS), in which the pre-S_1 _and -S_2 _regions of the LHBS gene are partially deleted, are highly associated with HBV-related HCC.

**Methods:**

The pre-S region of the LHBS gene in two hundred and one HBV-positive serum samples was PCR-amplified and sequenced. A pre-S oligonucleotide gene chip was developed to efficiently detect pre-S deletions in chronic HBV carriers. Twenty serum samples from chronic HBV carriers were analyzed using the chip.

**Results:**

The pre-S deletion rates were relatively low (7%) in the sera of patients with acute HBV infection. They gradually increased in periods of persistent HBV infection: pre-S mutation rates were 37% in chronic HBV carriers, and as high as 60% in HCC patients. The Pre-S Gene Chip offers a highly sensitive and specific method for pre-S deletion detection and is less expensive and more efficient (turnaround time 3 days) than DNA sequencing analysis.

**Conclusion:**

The pre-S_1/2 _mutants may emerge during the long-term persistence of the HBV genome in carriers and facilitate HCC development. Combined detection of pre-S mutations, other markers of HBV replication, and viral titers, offers a reliable predictive method for HCC risks in chronic HBV carriers.

## Background

Chronic hepatitis B virus infection is a major cause of HCC worldwide and its most important cause in Asia [1-6]. HBV infection occurs primarily through blood or body fluid transmission. HBV-related HCC often occurs at the age of 40 or older, suggesting that HBV may persist in carriers for decades before HCC actually develops [6,7]. Long-term monitoring of chronic HBV carriers is important to help prevent HCC. Furthermore, implementing cancer therapies at early disease stages is beneficial. The HBV markers commonly used to monitor the viral status in chronic HBV carriers are viral DNA titers, HBV surface and core antigens, and hepatitis B envelope (HBe) antigen [7,8]. Combined detection of these markers reveals the status of virus replication as well as the number of virus particles in host hepatocytes. Active HBV viral replication and high virus titers are associated with the severity of HBV-induced liver inflammation, fibrosis, cirrhosis, and HCC [9,10].

**Figure 1:**
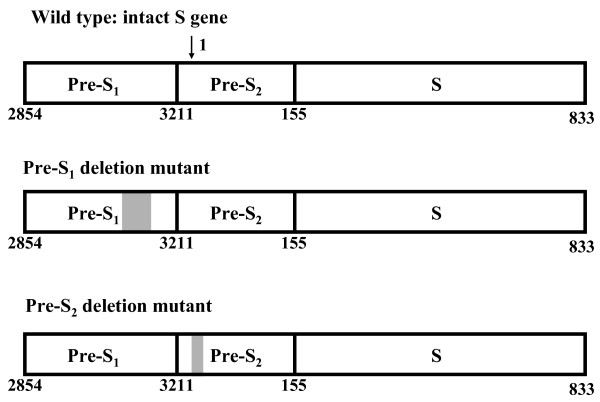
**Representatives of the wild-type, pre-S_1_, and pre-S_2 _mutant LHBS gene**. The shaded boxes are the regions deleted in the pre-S_1 _and pre-S_2 _mutant LHBS. The numbers on the bottom of the gene indicate the pre-S_1_, S_2_, and S regions of the LHBS gene in nucleotide order. Nucleotide 1 is the start of the circular genome and the numbers go clockwise. The last nt number is 3221. Here only the S gene, which spans the start of the genome, is shown. The arrow at the top of the diagram indicates the start (nt 1) of the HBV genome.

In the late 1990s, two major types of pre-S deletion mutant LHBS were identified and highly associated with HCC [11,12]. LHBS is expressed primarily at the late stage of chronic HBV infection, after the viral genome has integrated into the host chromosome [13-16]. Pre-S mutant LHBS was first isolated from ground-glass hepatocytes (GGH), the histological hallmarks of the late stages of chronic HBV infection, and is often seen in the liver sections of HCC patients [17]. Pre-S mutant LHBS is highly associated with advanced liver diseases, including cirrhosis and HCC, which suggests that it contributes to hepatocellular carcinogenesis [18-26]. In the two types of pre-S mutant LHBS, pre-S_1 _and pre-S_2 _mutant LHBS, the pre-S_1 _and -S_2 _regions are, respectively, partially deleted (Fig. 1) [11,12]. They accumulate in endoplasmic reticulum (ER) and induce strong ER stress [27]. Through an ER stress-mediated pathway, they cause oxidative stress and DNA damage [28]. Through an ER stress-independent pathway, however, pre-S_2 _mutant LHBS contributes to the increased proliferation of hepatocytes [29]. Pre-S_2 _mutant LHBS also interacts with c-Jun activation domain-binding protein 1 (JAB1) and induces p27^Kip1 ^degradation and cell-cycle progression [30]. Through an unknown mechanism, pre-S_2 _mutant LHBS also induces significant cyclin A expression [29]. Based on the findings of these previous studies, pre-S mutant LHBS, especially the pre-S_2 _type, is believed to be crucial in HBV-associated hepatocellular carcinogenesis.

After pre-S mutant LHBS was discovered, various geographically diverse studies [18-26] screening for pre-S mutations invariably reported that they were prevalent in chronic HBV carriers. In addition, pre-S mutant LHBS, especially the pre-S_2 _type, is highly correlated with the severity of HBV-related liver diseases, including HCC [20-23,25]. Therefore, it is important to screen for pre-S deletion mutations in chronic HBV carriers. This type of screening should be done in combination with the detection of other HBV markers, such as viral titers and HBe antigen (Ag), to estimate an HBV carrier's relative risk for HCC.

Identifying pre-S mutations in chronic HBV carriers usually requires multiple experimental procedures, because most pre-S mutants co-exist with wild-type LHBS in blood and hepatocytes; this is probably due to the emergence of the pre-S mutant from the wild-type form. One individual carrier often simultaneously presents multiple pre-S mutant forms that need to be singly cloned and then sequenced. This is both time-consuming and expensive, which makes it difficult to screen large populations of chronic HBV carriers. We developed a convenient and cost-effective oligonucleotide array system for screening pre-S deletions in the LHBS gene. Using this system, we omitted traditional DNA sequencing and shortened the detection procedure from about 7 days to no longer than 3 working days.

## Materials and methods

### Reagents and participants

The pre-S region of the LHBS gene in two hundred and one HBV-positive serum samples, collected at the National Cheng Kung University Hospital from 2000 through 2002, was PCR-amplified and sequenced [31]. To develop the Pre-S Gene Chip system, 20 HBV-DNA-positive serum samples were obtained from Kung-Chia Young, PhD, at National Cheng Kung University. The pre-S region of the LHBS gene was PCR-amplified and sequenced [31]. The DNA sequences were used to design oligonucleotide probes on the Pre-S Gene Chip. To test the efficacy of the chip, another 20 serum samples from chronic HBV carriers were obtained from the Center for Disease Control, Taipei, Taiwan, and analyzed using the chip. A DNA extraction kit (QIAamp MinElute Virus Spin kit; Qiagen Inc., Valencia, CA) was used to extract the virus DNA in serum. Most of the common chemicals used for chip hybridization were purchased from Sigma-Aldrich Co., St Louis, MO.

### The Pre-S Gene Chip

**Figure 2:**
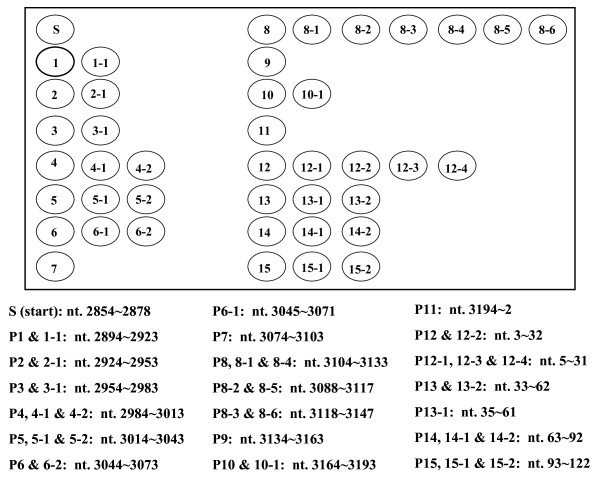
**The Pre-S Gene Chip**. The chip (7 mm (H) × 10 mm (W)) contains 42 oligonucleotide probes spanning the pre-S regions. The target region of each probe is indicated in the order of the nucleotides below the chip. The probes with extension numbers (e.g., 8-1, 8-2,... 8-6) are the redundant probes that target the same regions as the primary probes (e.g., 8) do.

The oligonucleotide DNA probe (20 μM) was prepared in DNA tracking dye (5 mM Tris-HCl, 0.5 mM Na_2_EDTA, 0.75 mg/mL bromophenol blue, 15% glycerol [pH 7.5]). The 42 DNA probes spanning the pre-S region (532 nucleotides (nt)) of the LHBS gene were spotted on positively-charged nylon membrane, using an arrayer (Ezspot Robotic Arrayer; EZlife Technology, Inc., Taipei, Taiwan) with a solid pin 400 μm in diameter. The probes ranged from 25 to 30 nt long, and some were linked with 15 to 17 thymidines at the 5' ends. Redundant probes were designed to target the genetically polymorphic regions in the gene (Fig. 2). After the probes had been spotted, the DNA on the membrane was ultravioletly (UV) cross-linked (Stratalinker UV Crosslinker; Stratagene, La Jolla, CA) to the nylon membrane.

### Polymerase chain reaction

**Figure 3:**
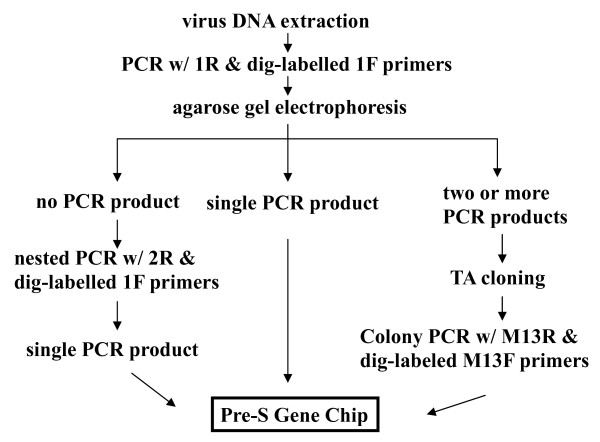
**Working scheme of Pre-S Gene Chip analysis**. The virus DNA is extracted from the patient's blood or liver tissue. The pre-S region is PCR-amplified using PCR-1R and 5'-dig-labeled 1F primers. The PCR products are visualized using agarose gel electrophoresis and ethidium bromide staining. In cases where no PCR products are seen---perhaps because of a low HBV DNA titer---nested PCR using PCR-2R and 5'-dig-labeled 1F primers is done. When only a single PCR product is seen, the DNA product is directly subjected to chip hybridization. However, when two or more different types of pre-S PCR products are seen in agarose gel, the products are first directed to TA cloning. Multiple plasmid clones are then analyzed using colony PCR with M13R and 5'-dig-labeled 13F primers. These PCR products are then analyzed using the Pre-S Gene Chip.

The pre-S LHBS gene was PCR amplified by using the primers 1F (digoxigenin labeled at the 5' end) and 1R (Table 1). In cases where no visible DNA products were seen using agarose gel electrophoresis because of low viral titers in the serum samples, nested PCR using the primers 1F and 2R was then done (Fig. 3). For *E. coli *colony PCR, the M13 Forward (digoxigenin labeled at the 5' end) and M13 Reverse primers (Invitrogen, Inc., Carlsbad, CA) were used. The PCR mix contained 10 ng of template DNA, 0.2 μM of each primer, 200 μM of each dNTP, 0.5 units of SuperTherm Fold DNA polymerase (JMR Holdings, Inc., Commerce Township, MI) with 1.5 mM of MgCl_2_, 25 mM of N- [Tris (hydroxymethyl) methyl]-3-aminopropanesulfonic acid (TAPS [pH 9.3]), 50 mM of KCl, 2 mM of MgCl_2_, 1 mM of β-mercaptoethanol, and 0.25 μg/μL of activated calf thymus DNA. Agarose gel electrophoresis was used to examined the PCR products.

**Table 1:** The PCR primers used in this study.

Primer	Sequence	Comment
Primer-1F	5'-GCGGGTCACCATATTCTTGGG-3'	HBV genome nt 2818 to nt 2837

Primer-1R	5'-GAGTCTAGACTCTGCGGTAT-3'	HBV genome nt 236 to nt 255

Primer-2R	5'-TAACACGAGCAGGGGTCCTA-3'	HBV genome nt 180 to nt 199

M13 Forward primer	5'-GTAAAACGACGGCCAGT-3'	

M13 Reverse primer	5'-AACAGCTATGACCATG-3'	

nt, nucleotide

### TA cloning

In cases where multiple pre-S PCR products were revealed, *E. coli *TA cloning was done. Three microliters of PCR product was ligated with 50 ng of TA cloning vector (pCR 2.1; Invitrogen) at 14°C overnight. The next day, the ligation product was transformed into an *E. coli *DH5α strain. The pre-S insert-DNA was then examined using colony PCR. For the DNA sequencing analysis, the colony PCR products were purified using a kit (PCR Clean-Up Kit; Roche Applied Sciences, Indianapolis, IN) and sent to our university's DNA Sequencing Facility for analysis.

### Pre-S Gene Chip analysis

The pre-S chip was placed in a 24-well culture plate, soaked in 0.2× standard sodium citrate (SSC: 150 mM NaCl, 15 mM NaH_2_(C_3_H_5_O(COO)_3 _[pH 7.0]), and then rinsed twice with the same solution. For pre-hybridization, the chip was then gently shaken in microarray hybridization buffer (5× SSC, 1% blocking reagent (Roche), 0.1% N-lauroylsarcosine, 0.02% SDS) at room temperature for 2 h. The chip was then hybridized with 10 μL of heat-denatured PCR product in 300 μL of hybridization buffer at 50°C for 90 min while being gently shaken (60 rpm). After it had been hybridized, the chip was washed four times with 0.1× SSC and then incubated in 1× blocking reagent at room temperature for 1 h. The digoxigenin that had hybridized to the DNA probes on the chip was then recognized by incubating the chip with 0.375 units of anti-digoxigenin-alkaline phosphatase Fab fragments (Roche) for 1 h. After it had been incubated, the chip was washed 3 times with MAB washing buffer (MAB: 0.1 M maleic acid, 0.15 M NaCl, 0.1% Tween 20 [pH 7.5]) for 15 min each. To detect digoxigenin signals, the chip was first soaked in a detection buffer (0.1 M Tris-HCl, 0.1 M NaCl [pH 9.5]) for 5 min and then incubated with two alkaline phosphatase substrates: nitro blue tetrazolium chloride (NBT, 0.375 mg/mL) and 5-bromo-4-chloro-3-indolyl phosphate (BCIP, 0.188 mg/mL) at 37°C for 20 min in the dark. Finally, the chip was washed 3 times with ddH_2_O and air-dried. Chip images were scanned using an Epson Perfection 1250 scanner [32].

## Results

Using DNA sequencing analysis, we first detected pre-S deletion mutations in the sera of HBV carriers and patients with HBV-related HCC. The pre-S mutation rate was relatively low (7%) in the sera of patients with high HBV titers, which indicates the acute phase of HBV infection (Table 2). The pre-S deletion rate gradually increased during periods of persistent HBV infection: the rate was 37% in HBV carriers with low serum HBV titers, which indicated the chronic phase of HBV infection. In HCC patients with HBV infection, the pre-S deletion rate was as high as 60%, which suggested that pre-S mutants emerge during long-term persistence of the HBV genome in carriers, and that they facilitate the development of HCC.

**Figure 4:**
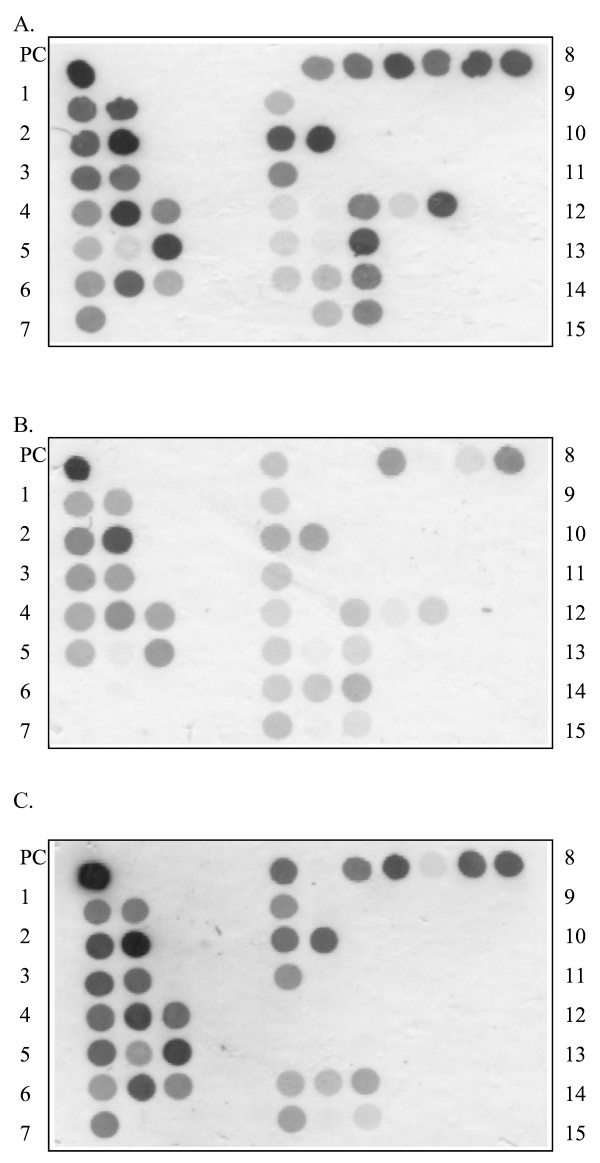
**Results of the Pre-S Gene Chip analysis**. **A**: The wild-type LHBS gene. **B**: The pre-S_1 _mutant LHBS, with nt 3044-3103 of the HBV genome deleted. The signals on probes 6 (nt 3045-3071) and 7 (nt 3074-3103) are negative. **C**: The pre-S_2 _mutant LHBS, with nt 3-57 of the HBV genome deleted. The signals on probes 12 (nt 3-32) and 13 (nt 33-62) are negative.

To efficiently detect pre-S deletion mutations, we developed a Pre-S Gene Chip on which contained 42 DNA probes that targeted the pre-S region of the LHBS gene (Fig. 2). To test the efficacy of the chip, we first used isolated plasmids containing wild-type HBV, pre-S_1 _mutant with nt 3044 to 3103 of the LHBS gene deleted, and pre-S_2 _mutant with nt 3 to 57 deleted [28]. These wild-type and pre-S mutant LHBS genes were initially isolated from genotype B HBV carriers in Taiwan [11,12]. The wild-type clone hybridized to all the DNA probes on the chip (Fig. 4). The pre-S_1 _and -S_2 _mutants, however, showed signals missing in probes 6 to 7, which spanned nt 3044 to 3103 in the pre-S_1 _region, and probes 12 to 13, which spanned nt 3 to 62 in the pre-S_2 _region, respectively. These results showed that the Pre-S Gene Chip correctly detected the pre-S deletions in the LHBS gene.

**Table 2:** Pre-S mutations in patients with different serum HBV DNA titers

Status	Serum HBV DNA Titer*	Cases (*n*)	HBV Clones^# ^(*n*)	Clones with Pre-S Deletions (*n*)	Prevalence
Hepatitis	High	53	57	4	**7.0%**
	
	Intermediate	62	72	10	**13.9%**
	
	Low	43	54	20	**37.0%**

HCC	-	43	73	44	**60.3%**

HCC, hepatocellular carcinoma.

*High titer: HBV genome ≥ 10^8 ^copies/mL serum; intermediate titer: HBV genome ≥ 10^6 ^and < 10^8 ^copies/mL serum; low titer: HBV genome < 10^6 ^copies/mL serum.

^#^The different pre-S HBS PCR products in one case were individually cloned and sequenced.

We then did a blind test on 20 cases to compare the pre-S typing results yielded by the Pre-S Gene Chip with those provided by classic DNA sequencing. Overall, the Pre-S Gene Chip yielded consistent pre-S deletion detection results with DNA sequencing (Table 3). Because the Pre-S Gene Chip used DNA hybridization signals to detect DNA deletions, the ranges spanning the deleted regions might have shifted a few nucleotides from the DNA sequencing results; however, that appeared not to have affected its identifying pre-S deletions in samples. In summary, DNA sequencing and Pre-S Gene Chip analyses were used to identify 21 and 19 pre-S deletion clones, respectively. The detection rates for pre-S deletions in these HBV carriers were 70% (14/20) for DNA sequencing and 65% (13/20) for the Pre-S Gene Chip. These data indicated that the Pre-S Gene Chip offered a sensitive assay compatible with DNA sequencing. We also found that 9 of the 14 pre-S mutant-positive cases had multiple pre-S products and that most (8/9) simultaneously contained wild-type as well as pre-S mutants. In addition, 4 cases had multiple mutation clones. This finding showed that wild-type and multiple pre-S mutant LHBS clones often co-existed in one individual.

**Table 3:** Summary of the pre-S genotyping results by DNA sequencing and Pre-S Gene Chip analysis in 20 HBV carriers.

Case	DNA Sequencing Result	Pre-S Gene Chip Result
1	wild-type	wild-type
2	1. wild-type	1. wild-type
	2. del nt 2894 to 3100	2. del nt 2894 to 3013 and nt 3074 to 3103
3	wild-type	wild-type
4	wild-type	wild-type
5	1. wild-type	1. wild-type
	2. del nt 3040 to 3111	2. del nt 3044 to 3103
6	wild-type	wild-type
7	del nt 3218 to 8	wild-type
8	1. wild-type	1. wild-type
	2. del nt 3084 to 3188	2. del nt 3074 to 3103 and nt 3134 to 3193
	3. del nt 3148 to 29	3. del nt 3134 to 32
	4. del nt 3138 to 59	4. del nt 3134 to 62
9	1. wild-type	1. wild-type
	2. del nt 3202 to 36	2. del nt 3194 to 32
10	del nt. 2913 to 3118 and nt 3217 to 9	del nt 2924 to 2983 and nt 3014 to 3103
11	Wild-type	wild-type
12	wild-type	wild-type
13	1. wild-type	1. wild-type
	2. del nt 3022 to 3205	2. del nt 3014 to 3103 and nt 3134 to 2
	3. del nt 3132 to 37	3. del nt 3134 to 32
	4. del nt 3057 to 3198	4. del nt 3074 to 3103 and nt 3134 to 3193
	5. del nt 3132 to 3182	5. del nt 3134 to 3193
14	del nt 2854 to 2871	del nt 2854 to 2878
15	1. del nt 3205 to 3213	1. wild-type
	2. del nt 34 to 54	2. del nt 33 to 62
16	1. wild-type	1. wild-type
	2. del nt 3041 to 3126	2. del nt 3044 to 3103
	3. del nt 25 to 56	3. del nt 33 to 62
17	1. wild-type	1. wild-type
	2. del nt 2984 to 3221	2. del nt 2984 to 3013, nt 3044 to 3103, and nt 3134 to 2
18	1. wild-type	1. wild-type
	2. del nt 3 to 56	2. del nt 3 to 62
19	del nt 3040 to 3111	del nt. 3044 to 3103
20	del nt 2948 to 3097 and nt 3112 to 2	del nt 2954 to 3013, nt 3044 to 3103, and nt 3194 to 2
**Pre-S mutant Clones (*n*)**	**21**	**19**
**Cases with Pre-S Mutations (*n*) (%)**	**14 (70%)**	**13 (65%)**

nt, nucleotide

## Discussion

In this study, we developed an oligonucleotide array system to detect deletions in the pre-S regions of the HBV LHBS gene in chronic carriers with detectable HBV DNA, the group of carriers at a high risk of HCC [10]. We found that multiple mutation clones often co-exist in a carrier, making genotyping tedious and time-consuming. Cloning individual PCR products is usually necessary to differentially genotype the different pre-S deletion products. We found that doing colony PCR of the individual gene clones, and then directly applying the PCR products to the Pre-S Gene Chip for hybridization and detection, was more time- and cost-effective than traditional DNA sequencing analysis. The Pre-S Gene Chip is made of a 0.7-cm^2 ^nylon membrane and costs substantially less than DNA sequencing analysis, especially for clinical institutions without internal access to a DNA sequencing service. The DNA chip system also simultaneously detects multiple pre-S clones, which makes it convenient for large-scale pre-S mutation screenings in chronic HBV carriers. Using the Pre-S Gene Chip and DNA sequencing in blind tests of 20 cases showed that both methods had close detection rates of pre-S deletions. Therefore, we conclude that the Pre-S Gene Chip delivered comparable results to direct DNA sequencing and is a good screening method for pre-S deletions. A minor disadvantage of the Pre-S Gene Chip system is that it does not appear to be sensitive enough to detect deletions less than 10 nt long: in one blind-tested case, the chip did not identify a 10-nt deletion clone. Nevertheless, considering cost and convenience, the Pre-S Gene Chip is potentially good for large-scale screening of pre-S deletions; perhaps it can be improved.

In this study, we found that pre-S deletion rates were significantly higher in the late phase of chronic HBV infection, which displays lower HBV titers, than in the acute infection phase, which displays high viral titers. In the HBV-induced HCC stage, the pre-S deletion rate was as high as 60%. These findings suggested that pre-S deletions probably occur during long-term persistent HBV infection and eventually become predominant in HBV carriers. Therefore, we hypothesize that pre-S mutants are important in liver carcinogenesis. Our earlier studies [27,28] found that pre-S mutant LHBS accumulated in ER, where it caused ER and oxidative stress [27,28]. It also strongly induced DNA damage and mutations, which destabilized the genome [28]. We found that the pre-S_2 _mutant LHBS induces cyclin A overexpression, cell cycle progression, and cell proliferation, all essential factors for carcinogenesis [29,30]. These earlier findings showed that pre-S mutant LHBS, especially the pre-S_2 _type, is involved in carcinogenesis. Identifying the pre-S deletion mutations in chronic HBV carriers is therefore important when screening for persons at high risk for HCC.

In addition to pre-S deletions, a number of HBV markers, such as HBV viral titers and HBeAg, are also believed to be associated with the risk of HCC in chronic HBV carriers [9,10]. HBV replication causes liver injury and inflammation, which releases cytokines and facilitates the development of fibrosis and liver cell proliferation [7,8]. In addition, the HBV protein HBX crosstalks with various host factors and behaves as a viral oncoprotein [33,34]. It transactivates a number of cellular promoters, acting on cis-acting regulatory elements [35]. It also regulates proteasome function and, thus, controls the degradation of cellular and viral proteins [36]. We hypothesize that pre-S mutant LHBS strengthens the detrimental effects of HBX protein by increasing cell proliferation and genomic instability, thereby facilitating hepatocellular carcinogenesis.

## Conclusion

We developed the Pre-S Gene Chip system to screen for pre-S deletions in the LHBS gene. The detection sensitivity of the Pre-S Gene Chip for pre-S mutations is close to that of direct DNA sequencing analysis, but it is much more time- and cost-effective. We believe that the Pre-S Gene Chip is feasible for the large-scale screening of pre-S mutations in chronic HBV carriers. Combining the detection of pre-S mutations with other HBV factors, such as HBV viral titers and HBeAg, should offer a reliable predictive method for HCC risk in chronic HBV carriers.

## Competing interests

A U.S. Provisional Patent (Title: *Oligonucleotides and use thereof for determining deletion in HBV pre-S region*. Application No. 61077522, 2008) has been filed for the Pre-S Gene Chip (Fan-Ching Shen, Ih-Jen Su and Wenya Huang). All of the other authors declare that they have no competing interests.

## Authors' contributions

FS carried out the pre-S chip preparation and the majority of molecular studies. IS conceived of the pre-S study project. HW supplied the PCR primer sequences and offered discussion for this study. YH participated in the PCR experiments. WY supplied the patient specimens for pre-S genotyping studies (Table 2). KY supplied control samples for probe design for the pre-S chip. TC offered advice and technical help for probe design for the pre-S chip. HH and HT participated in the evaluation of efficiency of the pre-S chip detection. WH designed the experiments and wrote the manuscript. All authors read and approved the final manuscript.
